# Risk factors for hospital re-presentation among older adults following fragility fractures: a systematic review and meta-analysis

**DOI:** 10.1186/s12916-016-0671-x

**Published:** 2016-09-12

**Authors:** Saira A. Mathew, Elise Gane, Kristiann C. Heesch, Steven M. McPhail

**Affiliations:** 1 School of Public Health & Social Work and Institute of Health and Biomedical Innovation, Queensland University of Technology, Brisbane, Australia; 2 Queensland Department of Health, Centre for Functioning and Health Research, Metro South Health, Brisbane, Australia; 3 School of Health & Rehabilitation Sciences, The University of Queensland, Brisbane, Australia

**Keywords:** Readmissions, Frailty, Fractures, Geriatric, Risk factors

## Abstract

**Background:**

Older adults hospitalized with fragility fractures are at high risk of negative events that can culminate in re-presentations to hospital emergency departments or readmissions to hospital. This systematic review aimed to identify patient, clinical, or hospital-related factors that are identifiable at the index admission and that may be associated with re-presentations to hospital emergency departments or hospital readmissions in older adults following fragility fractures.

**Methods:**

Four electronic databases (PubMed, CINAHL, Embase, and Scopus) were searched. A suite of search terms identified peer-reviewed English-language articles that examined potential correlates of hospital re-presentation in older adults (mean age ≥ 65 years) who were discharged from hospital following treatment for fragility fractures. A three-stage screening process (titles, abstracts, full text) was conducted by two researchers independently. Participant characteristics, study design, potential correlates examined, analyses, and findings were extracted for studies included in the review. Quality and risk of bias were assessed with the Effective Public Health Practice Project Quality Assessment Tool. The strength of evidence was incorporated into a best evidence synthesis, and meta-analysis was conducted where effect pooling was possible.

**Results:**

Eleven of 35 eligible studies were categorized as high quality studies. These studies reported that age, higher Cumulative Illness Rating scores, American Society of Anesthesiologists scores > 3, longer length of stay, male sex, cardiovascular disease, low post-operative hemoglobin, kidney disease, dementia and cancer were factors identified at the index admission that were predictive of subsequent re-presentation to hospital. Age was the only predictor for which pooling of effects across studies was possible: pooling was conducted for re-presentation ≤ 30 days (pooled OR, 1.27; 95 % CI, 1.14–1.43) and > 30 days (pooled OR, 1.23; 95 % CI, 1.01–1.50).

**Conclusions:**

The best-evidence synthesis, in addition to the meta-analysis, identified a range of factors that may have utility in guiding clinical practice and policy guidelines for targeted interventions to reduce the need for re-presentation to hospital among this frail clinical population. The paucity of studies investigating re-presentations to hospital emergency departments without admission was an important gap in the literature identified in this review. Key limitations were exclusion of non-English language studies and grey literature.

**Systematic review registration:**

PROSPERO CRD42015019379.

## Background

The incidence of fragility fractures is expected to rise as the population of older adults increases [1, 2]. Fragility fractures are fractures sustained from relatively minor forces (e.g., fall from standing height or less) and often occur among people with osteoporosis [3]. Negative outcomes associated with these fractures include disability, morbidity, hospitalization, and increased risk of premature death following the fracture event [4]. These unfavorable outcomes burden patients and increase demand on healthcare services [5, 6].

During an index presentation to hospital after a fragility fracture, the fracture will be examined, and unstable fractures will typically be stabilized using either surgical or non-surgical approaches [7]. Following acute management of the fracture and potentially inpatient rehabilitation, patients are discharged from hospital. However, a re-presentation to hospital may be required soon after discharge [8].

Although there is inconsistency regarding time-frames between studies investigating hospital re-presentations, these may typically be considered to include subsequent unplanned visits to a hospital sometime within the first 2 years following hospitalization [9]. They include emergency department (ED) visits without hospital admission, same-day discharges, and inpatient admissions for 1 or more days. Most older adults returning to hospital within 1 month re-present with a clinical problem or diagnosis related to their index admission, and this is a relatively frequent occurrence among older adults [10].

For those seeking to decrease re-presentation rates after treatment for fragility fractures, it is advantageous to understand the factors that predict re-presentations. To date, no systematic review has examined the range of reported risk factors for hospital re-presentation among older adults following hospitalization for fragility fracture management. One systematic review examined the timing of surgery on negative outcomes following hip fractures [11]. The authors concluded that surgery within 48 hours of hospital admission for a hip fracture reduced the length of hospital stay, mortality rates, and complications. They also concluded that surgical delays increased the risk of complications. Another review examined the outcomes of patients with osteoporotic fractures after hospital discharge [12]. Those patients were reported to be at high risk of morbidity, mortality, and subsequent fracture. Another systematic review summarized the risk factors for hospital readmissions in non-fracture-specific samples and reported that functional disability and comorbidities were correlated with readmission to hospital [13].

Research findings summarized in the aforementioned reviews provide some understanding of the risk of negative outcomes after hospital discharge that may have relevance to people recovering from fragility fractures. However, people recovering from fragility fractures may not have the same risk profile as those who are less frail or admitted to hospital for other health conditions. Therefore, the aim of the present study was to examine potential correlates of hospital re-presentation following fragility fractures in older adults. Specifically, the review focused on reports of patient-, clinical-, or hospital-related factors that could be identified at the time of the initial hospitalization, and re-presentation time-frames of up to 2 years after the initial hospitalization.

## Methods

### Design

The protocol for this systematic review and meta-analysis has previously been reported and is summarized below [14].

#### Data sources and searches

Databases were searched for articles in peer-reviewed, English-language journals from the onset of the databases until August 24, 2015. The literature was searched in phases. First, a comprehensive list of terms and synonyms of re-presentations, fracture, elderly, and hospital were combined with Boolean operators to formulate a search string. Second, a systematic search was conducted using the search string to identify relevant studies in four electronic databases: EMBASE, PubMed/Medline, Scopus, and CINAHL via the EBSCO interface. The search strings adapted for each database are presented in Table 1. Finally, the reference lists of included articles were searched for additional relevant studies. Studies identified through reference lists were initially assessed for relevance by study title and abstract. The results were imported into reference management software (Endnote) to manage, extract data and delete duplicate references.

**Table 1:** Search syntaxes customized for each database

Database	Search syntax
PubMed	(fracture[MeSH Terms]) AND (((readmi* or rehosp* or re-admi* or re-hosp* or re-presentation)) OR “Patient Readmission”[MeSH]) Filters: Aged: 65+ years
CINAHL	“fracture* AND (readmi* or rehosp* or re-admi* or re-hosp* or re-presentation) Age Groups: Aged: 65+ year
Embase	“fracture”/exp and (readmi* or rehosp* or re-admission or re-hospitalisation or re-hospitalization or re-presentation) AND ([aged]/lim OR [very elderly]/lim)
Scopus	ABS fracture* AND (readmi* OR rehosp* OR re-admission OR re-hospitalisation OR re-hospitalization or re-presentation) AND (aged OR elderly OR geriatric OR old*)

#### Study selection

The selection of articles consisted of three stages of screening (titles, abstracts, full text), which were conducted by SAM and EG independently of each other. A third author (SMM) arbitrated any unresolved disagreements arising during any stage in the search and screening process. Further details about the search and selection strategy were outlined in the protocol [14].

##### Types of studies

Quantitative studies that explored the correlates of hospital re-presentations in older adults for any time-frame within the first 2 years were eligible for inclusion. Both epidemiological (retrospective and prospective cohort studies) and experimental study designs (that also reported risk factors from analyses of participating cohorts) were eligible for inclusion. Cohort studies were classified as retrospective if the hospital re-presentations had already occurred at the time of study planning and historical cases or events were being audited. In contrast, cohort studies were classified as prospective if study planning occurred prior to the study enrolment period in which hospital re-presentations were observed. Qualitative studies and grey literature were excluded. Authors of included studies were contacted for further information.

##### Types of participants

Only studies that recruited older adults (mean age ≥ 65 years) who were hospitalized following fragility fractures were included. There were no sex, race, ethnicity, residential status (residential care facilities, or elsewhere in the community), or socioeconomic status restrictions for participants.

##### Types of outcomes

Studies that examined hospital re-presentation as an outcome were included. Studies that examined correlates of re-presentations in a general patient population but reported separate analyses for re-presentations in older adults with fragility fractures were eligible for inclusion. Outcomes of secondary interest were the number and frequency of re-presentations, the rate of re-presentations, and days since discharge to re-presentation.

#### Data extraction and quality assessment

Two reviewers conducted the data extraction and quality assessment independently (SAM and EG). A third reviewer (SMM) arbitrated unresolved disagreements. The data extracted included details about the participant characteristics, study design, inclusion and exclusion criteria, risk factors, primary outcomes (re-presentations), and statistical analysis. The quality of individual studies and risk of bias were assessed with the Effective Public Health Practice Project Quality Assessment Tool [15, 16]. This quality assessment tool can be widely used to rate the methodological parameters across all quantitative study designs. A best-evidence synthesis was implemented to integrate the strength of evidence of studies [17].

#### Data synthesis and analysis

Substantial methodological, statistical, and quality of reporting heterogeneity present in the studies was considered by the investigators to prohibit the valid pooling of effects (meta-analysis) for all potential predictors except age. Age was the only factor for which the definition and method of reporting results were somewhat similar across a pool of studies. Hence, the extracted study characteristics and results from all eligible studies were tabulated and summarized in a best evidence synthesis, and a meta-analysis was performed to obtain pooled estimates for age for re-presentations within 30 days and re-presentations after 30 days using RevMan (version 5.1, Cochrane Collaboration).

For the meta-analysis, odds ratios (ORs) were not able to be directly obtained in a consistent and easily interpretable format (e.g., estimates of effect per increasing year of age) due to differences in statistical analyses and reporting among studies that included age as a potential correlate of hospital re-presentation. To obtain ORs from each study, the following strategy was used. First, effect sizes (ORs, relative risks or hazard ratios) were extracted or calculated from original studies where possible. Because some studies reported effect sizes for age separately for different subgroups, the effect sizes for these groups were merged via inverse variance pooling before entering them into the meta-analysis. If ORs and confidence intervals (CI) were reported, these were taken directly from the studies. If ORs were reported separately for different re-presentation time periods within a study, the results were combined (with meta-clustering) to give one estimate for re-presentation within 30 days, and one estimate for re-presentation after 30 days [18]. If relative risks were reported, prevalence of the risk factors and incidence of hospital re-presentations were used to calculate ORs from available data. Rate ratios and standardized mean differences were extracted and calculated from *P* values to calculate ORs, where relevant. The random effects model of analysis was used to best account for heterogeneity, and tests of heterogeneity (I^2^) were performed. A sensitivity analysis was performed to examine the effect of removing one small study [19] with an age effect estimate for re-presentation within 30 days that fell outside the confidence ranges of any other included studies (OR estimate was considerably higher).

## Results

**Fig. 1:**
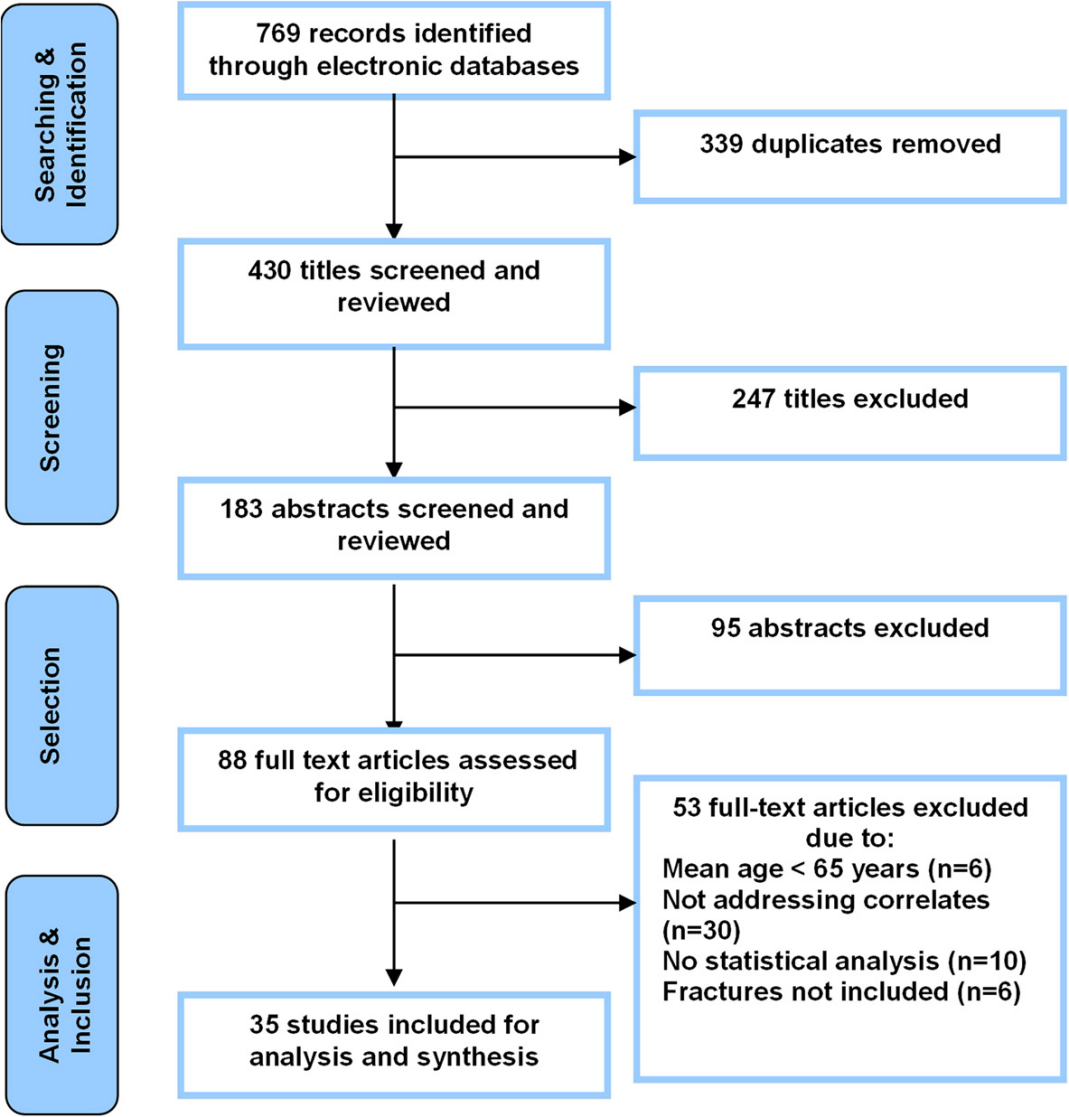
Study selection flow diagram

The outcome of the study identification and selection process is outlined in Fig. 1. In summary, after the removal of 339 duplicates, a total of 430 unique studies were identified across four databases. Eighty-eight articles were deemed eligible for full text screening, of which 53 studies were excluded for not meeting the inclusion criteria. The remaining 35 studies were included in this review.

### Study characteristics

The characteristics of the included studies are described in Table 2. The review included one randomized controlled trial that reported the effect of cholecalciferol and physiotherapy on hospital readmissions, but also reported correlates of re-presentation [20]. The remaining 34 studies were retrospective cohort studies (*n* = 23), prospective cohort studies (*n* = 9), an interrupted time series study (*n* = 1) [21], or a combination of retrospective and prospective cohort designs (*n* = 1) [22]. Despite the delineation between retrospective and prospective cohort studies in this review, it is perhaps noteworthy that both types typically used information sources recorded at (or at least near) the time of the events of interest (e.g., in patient medical records). Subsequently, the authors of this review did not consider there to be a substantial difference in interpretation of the reliability of data originating from the included retrospective and prospective studies. All studies addressed risk factors for hospital readmissions; none addressed risk factors for hospital re-presentations more broadly, which could have included ED presentations without admission to hospital. Therefore, below, only factors associated with readmissions are presented.

**Table 2:** Characteristics of studies included in the systematic review

Author and year of publication	Country	Site	Study design	Sample characteristics	Sample size/population	Study time period	Fracture site
Basques et al. (2015) [30]	USA	370 hospitals	Retrospective cohort	>70 years	8434	2011–2012	Hip
Bischoff-Ferrari et al. (2010) [20]	Switzerland	Single hospital	Randomized controlled trial	≥65 years	173	2005–2007	Hip
Boddaert et al. (2014) [21]	France	Single hospital	Interrupted time series	≥70 years	334	2005–2012	Hip
Fox et al. (1998) [31]	USA	8 hospitals	Prospective cohort	≥65 years	306	1990–1991	Hip
French et al. (2008) [25]	USA	Veterans Health Administration Medical Centre	Retrospective cohort	≥65 years	41,331	1999–2003	Hip
Giusti et al. (2008) [32]	Italy	Single hospital	Prospective cohort	≥70 years	236	2000–2001	Hip
Golinvaux et al. (2014) [48]	USA	350 hospitals	Retrospective cohort	≥65 years	9938	2005–2012	Hip
Gregersen et al. (2011) [42]	Denmark	Single hospital	Prospective cohort	≥65 years (Nursing Home Residents)	233	2006–2010.	Hip
Hageman et al. (2014) [43]	USA	Level 1 trauma center	Retrospective cohort	Mean age > 65	890	2008–2011	Hip
Halm et al. (2003) [27]	USA	4 hospitals	Prospective cohort	Mean age > 65	559	1997–1998	Hip
Halm et al. (2003) [33]	USA	4 hospitals	Prospective cohort	Mean age > 65	551	1997–1998	Hip
Halm et al. (2004) [34]	USA	4 hospitals	Prospective cohort	Mean age > 65	550	1997–1998	Hip
Härstedt et al. (2015) [35]	Sweden	Single hospital	Prospective cohort	Mean age > 65	272	2009–2011	Hip
Heidari et al. (2012) [26]	UK	62 hospital pharmacies	Retrospective cohort	Mean age > 65	255,841	2003–2007	Hip
Heyes et al. (2015) [29]	Ireland	Single hospital	Prospective cohort	Mean age > 65	451	2010–2012	Hip
Hsaio et al. (2011) [23]	Taiwan	Health insurance database	Retrospective cohort	Mean age > 65 (women)	11,278	2001–2007	Hip/Vertebra
Intrator and. Berg (1998) [44]	USA	Medicare beneficiaries	Retrospective cohort	≥70 years	324	1987–1991	Hip
Jou et al. (2014) [24]	Taiwan	Health insurance database	Retrospective cohort	Mean age > 65 (women)	9467	2003–2006	Hip
Kates et al. (2014) [28]	USA	Level 3 trauma center	Retrospective cohort	≥65 years	1081	2005–2010	Hip
Kates et al. (2015) [49]	USA	Level 3 trauma center	Retrospective cohort	≥65 years	1081	2005–2010	Hip
Khan et al. (2012) [36]	UK	Single hospital	Retrospective cohort	Mean age > 65	467	2009–2010	Hip
Kiel et al. (1994) [45]	USA	43 nursing homes	Prospective cohort	Mean age > 65	2624	1984–1988	Hip
Le-Wendling et al. (2012) [37]	USA	Single hospital	Retrospective cohort	≥65 years	308	2006–2008	Hip
Ling et al. (2013) [19]	Singapore	Single hospital	Retrospective cohort	Mean age > 65	254	2009–2010	Hip
Merchant et al. (2005) [38]	Singapore	Single hospital	Retrospective cohort	Mean age > 65	180	2001–2001	Hip
Ottenbacher et al. (2003) [46]	USA	171 hospitals	Retrospective cohort	Mean age > 65	9956	1994–1998	Hip
Pollock et al. (2015) [50]	USA	Level 1 trauma center	Retrospective cohort	Mean age > 65	1482	2005–2012	Hip
Radcliff et al. (2008) [51]	USA	Veterans Health Administration Medical Center	Retrospective cohort	Mean age > 65	5683	1998–2003	Hip
Riggs et al. (2010) [39]	USA	Single hospital	Retrospective cohort	≥65 years	606	2004–2006	Hip
Teixeira et al. (2009) [40]	France	Single hospital	Retrospective cohort	≥70 years	5709	2005–2006	Hip
Toson et al. (2015) [53]	Australia	247 hospitals	Retrospective cohort	Mean age > 65	47,698	2001–2010	Hip
Toy et al.(2014) [41]	USA	370 hospitals	Retrospective cohort	≥65 years	850	2011–2012	Vertebra
Tsai et al. (2013) [47]	Taiwan	National Health Insurance	Retrospective cohort	≥70 years	9238	2004–2007	Vertebra
Vochteloo et al. (2011) [22]	Netherlands	450 hospitals	Retrospective and prospective cohort	≥65 years	1222	2005–2010	Hip
Zhang et al. (2014) [52]	USA	State Inpatient Database	Retrospective cohort	Mean age > 65	27,017	2005–2010	Proximal humerus

Approximately half of the studies (*n* = 19, 54 %) were from the United States, with the remainder conducted in France (*n* = 2), Singapore (*n* = 2), Taiwan (*n* = 2), or elsewhere (*n* = 9). Sixteen studies (46 %) specifically targeted patients aged ≥ 65 years, although all reported a mean age > 65 years. Two studies included women only [23, 24]. One study restricted the analyses to nursing home residents [25].

The sample size of studies ranged from 173 patients [20] to 255,841 patients [26]. There were two kin studies that investigated different risk factors from the same large dataset [27, 28]. The total length of the enrollment period for the studies ranged from ≤ 2 years for 15 studies [27, 29–41], 3–5 years for eight studies [20, 22, 24–26, 42–47], 6–8 years for eight studies [21, 23, 28, 30, 48–52], and up to 10 years for one study [53]. This review focused on findings reported for re-presentations within the first 2 years after the index hospital event. Specifically, the observed timeframe for hospital re-presentations for findings reported in this review extended from 7 days to 18 months after the index hospital event [24, 31]. Hip fracture was the most common fracture site (*n* = 32 studies) [20–40, 42–46, 48–51, 53]. Two studies examined patients with vertebral fractures, and one study examined patients with proximal humerus fractures [41, 47, 52].

### Risk factors associated with re-presentations

The risk factors for hospital re-presentations that were examined are listed in Table 3 by shortest to longest observation time-frame after the index event in which re-presentation may have occurred. Most studies examined correlates of readmission within 30 days of the index event (i.e., 30 days since the initial hospital discharge (*n* = 8), an operation (*n* = 6), or admission to a nursing home (*n* = 1)). Other studies examined correlates within 60 days (*n* = 3), 90 days (*n* = 3), 6 months (*n* = 2), and 1 year (*n* = 7) from the index event. Two studies used multiple follow-up periods [24, 47]. For the purpose of this synthesis, correlates were categorized into patient characteristics and other clinical or hospital indicators.

**Table 3:** Reported associations between patient or clinical characteristics with risk of hospital re-presentations

Study	Patient characteristics	Association	Clinical/service characteristics	Association	Percentage of re-presentations
**Readmission** ^**a**^ **within 30 days**					
***Readmission within 7 days from discharge***					
**Tsai (2013)** [47]	Hospitalization for all reasons	OR = 0.48 (0.32–0.72)	Not investigated		Hospitalization for all reasons: 3.44 %
	Fracture related diagnoses	OR = 0.28 (0.12–0.68)			Fracture related diagnoses: 0.69 %
	Musculoskeletal disorder	OR = 0.08 (0.01–0.88)			Musculoskeletal disorders: 0.20 %
	Hospitalization for other diagnoses	OR = 0.67 (0.41–1.09)			Hospitalization for other diagnoses: 2.55 %
***Readmission within 14 days from discharge***					
**Jou et al. (2014)** [24]	14 days:		14 days: Medical center	Referent	50–74 y (3.21 %)
	Age < 75 Age ≥ 75	Referent	Regional hospital District hospital	**HR = 1.56 (1.08–2.25)**	
		**HR = 1.36 (1.08–1.71)**		**HR = 4.47 (3.20–6.26)**	
	14 days: CCI score 0 CCI score ≥ 2		LOS ≤10 days ≥11 days		≥75 y (4.16 %)
		Referent		Referent	
		**HR = 1.52 (1.22–1.92)**		HR = 0.25 (0.19–0.34)	
			14 days: Geographic regions		
			Northern Central Southern Eastern	Referent	
				HR = 1.21 (0.89–1.64)	
				HR = 1.17 (0.89–1.54)	
				HR = 0.96 (0.47–1.96)	
***Readmission within 28 days from discharge***					
**Khan et al. (2012)** [36]	Age	**OR = 1.06 (1.02–1.10)**	Not investigated		11 %
	Diabetes	**OR = 3.34 (1.54–7.25)**			
	History of neurological disorders	**OR = 5.66 (2.79–11.47)**			
	Admission other than home	**OR = 2.36 (1.19–4.66)**			
***Readmission within 30 days from discharge***					
**Boddaert et al. (2014)** [21]	CIRS score	**RR = 1.08 (1.00–1.16)**	Intervention vs. control group	**RR = 0.40 (0.23–0.70)**	Orthopedic group (usual care) = 17 %
	Age	RR = 0.99 (0.95–1.03)			Geriatric group (intervention) = 5 %
	Male sex	RR = 0.76 (0.41–1.41)			
**French et al. (2008)** [25]	Chronic heart failure	**OR = 1.24 (1.16–1.33)**	Inpatient LOS	**OR = 1.01 (1.01–1.02)**	18 %
	Cardiac arrhythmias	**OR = 1.11 (1.04–1.17)**			30 % occurred in the first week
	Other neurological disorder	**OR = 1.15 (1.05–1.26)**			60 % within 2 weeks
	Chronic pulmonary disease	**OR = 1.33 (1.25–1.40)**			81 % within 3 weeks
	Diabetes mellitus without chronic complication				
		**OR = 1.32 (1.15–1.52)**			
	Renal failure	**OR = 1.43 (1.29–1.60)**			
	Coagulopathy	**OR = 1.33 (1.16–1.52)**			
	Weight loss	**OR = 1.24 (1.07–1.44)**			
	Fluid and electrolyte disorders	**OR = 1.11 (1.04–1.20)**			
	Deficiency anemia	**OR = 1.16 (1.09–1.25)**			
	Alcohol abuse	OR = 0.86 (0.75–0.98)			
	Psychosis	OR = 1.16 (1.00–1.34)			
	Depression	OR = 1.06 (0.95–1.18)			
**Heidari et al. (2012)** [26]	Not investigated		Hospital drug policy for chemical thromboprophylaxis		55 %
			Aspirin	OR = 1.03 (0.87–1.23)	
			Heparin drug policy	OR = 1.06 (0.97–1.16)	
			Low-dose heparin	OR = 1.09 (0.93–1.28)	
**Jou et al. (2014)** [24]	30 days:		30 days: Medical center	Referent	50–74 y (3.21 %) ≥75 y (4.87 %)
	Age < 75 Age ≥ 75	Referent	Regional hospital District hospital	**HR = 1.51 (1.10-2.09)**	
		**HR = 1.34 (1.07–1.62)**		**HR = 3.82 (2.83–5.14)**	
	30 days:		LOS		
	CCI score 0 CCI score ≥ 2	Referent	≤10 days ≥11 days	Referent	
		**HR = 1.60 (1.30–1.97)**		HR = 0.32 (0.25–0.41)	
			30 days: Geographical regions		
			Northern Central Southern Eastern	Referent	
				HR = 1.25 (0.94–1.67)	
				HR = 1.20 (0.93–1.54)	
				HR = 1.00 (0.52–1.92)	
**Kates et al. (2014)** [28]	Age > 85	**OR = 1.52 (1.02–2.26)**	Time to surgery > 24 h	OR = 1.50 (1.00–2.25)	11 %
	CCI ≥ 4	**OR = 1.70 (1.02–2.81)**			
	Delirium	**OR = 1.65 (1.13–2.40)**			
	Dementia	**OR = 1.61 (1.12–2.33)**			
	History of arrhythmia with pacemaker	**OR = 1.75 (1.11–2.76)**			
	Placement presence of a pre-op arrhythmia	**OR = 1.62 (1.09–2.39)**			
	Partial or complete disability with ADL	**OR = 1.54 (1.05–2.26)**			
**Kates et al. (2015)** [49]	Age > 85	**OR = 1.58 (1.02–2.26)**	Not investigated		11.9 %
	Male	OR = 1.49 (1.00–2.24)			
	Assisted living	OR = 1.52 (0.82–2.59)			
	Skilled nursing	OR = 1.24 (0.84–1.85)			
	Parker mobility medium (5–8)	OR = 1.81 (0.98–3.35)			
	Parker mobility low (0–4)	OR = 1.50 (0.85–2.64)			
	Charlson score medium (2–3)	OR = 1.51 (1.03–2.25)			
	Charlson score high (4 or more)	OR = 1.65 (1.00–2.74)			
	Partial or complete disability	**OR = 1.51 (1.03–2.25)**			
	Delirium	**OR = 1.66 (1.14–2.41)**			
	Preoperative arrhythmia	**OR = 1.62 (1.09–2.39)**			
	Hematoma	OR = 7.51 (0.47–1.21)			
	Urinary tract infection	OR = 1.84 (0.39–8.84)			
	Pacemaker	**OR = 1.75 (1.11–2.76)**			
	Diabetes	**OR = 1.91 (1.22–2.99)**			
	Dementia	**OR = 1.61 (1.12–2.22)**			
	GERD	OR = 1.44 (0.99–2.10)			
	Cardiac disease	OR = 1.02 (0.66–1.59)			
	Alcoholism	OR = 1.12 (0.46–2.68)			
	Tobacco use	OR = 0.99 (0.56–1.73)			
**Le-Wendling et al. (2012)** [37]	Not investigated		Local vs. general anesthetic	OR = 2.0 (1.0-4.0)	19 %
**Pollock et al. (2015)** [50]	Pre-existing pulmonary disease	**OR = 1.88 (1.30–2.72)**	Discharge to skilled nursing facility	**OR = 1.5 (1.04–2.14)**	9 %
			Hospital LOS > 8 days	**OR = 1.88 (1.30–2.72)**	
**Toson et al. (2015)** [53]	Myocardial infarction	OR = 1.1 (1.0–1.2)	Not investigated		16 %
	Congestive heart failure	**OR = 1.2 (1.1–1.3)**			
	Peripheral vascular disease	OR = 1.2 (1.0–1.3)			
	Cerebrovascular accident	OR = 1.1 (1.0–1.2)			
	Dementia	OR = 0.8 (0.8–0.9)			
	Chronic pulmonary disease	OR = 1.1 (1.0–1.2)			
	Connective tissue disorder	OR = 1.2 (1.0–1.4)			
	Peptic ulcer	OR = 1.2 (1.0–1.5)			
	Mild liver disease	OR = 1.3 (1.0–1.7)			
	Diabetes without chronic complications				
		OR = 1.1 (1.0–1.2)			
	Diabetes with chronic complications	**OR = 1.2 (1.1–1.3)**			
	Hemiplegic or paraplegia	OR = 0.9 (0.8–1.1)			
	Renal disease	**OR = 1.3 (1.2–1.5)**			
	Any malignancy	**OR = 1.4 (1.2–1.6)**			
	Metastatic solid tumor	OR = 1.1 (0.9–1.4)			
	Moderate or severe liver disease	OR = 5.0 (3.3–7.5)			
**Readmission within 30 days post-operative**					
**Basques et al. (2015)** [30]	Age ≥ 90	**OR = 1.35 (1.09–1.67)**	Discharge to a facility	**OR = 1.42 (1.08–1.86)**	10 %
	Male	**OR = 1.40 (1.20–1.63)**	ASA class 3	**OR = 1.40 (1.09–1.69)**	
	BMI ≥ 35	**OR = 1.73 (1.24–2.44)**	ASA class 4	**OR = 1.90 (1.44–2.51)**	
	History of pulmonary disease	**OR = 1.46 (1.22–1.75)**			
	Hypertension	**OR = 1.21 (1.02–1.45)**			
	Steroid use	**OR = 1.38 (1.04–1.83)**			
	Partially dependent functional status	**OR = 1.31 (1.11–1.54)**			
	Fully dependent functional status	**OR = 1.41 (1.01–1.97)**			
**Golinvaux (2014)** [48]	Non-insulin dependent diabetes mellitus		Not investigated		Without diabetes = 5 %, Non-insulin dependent diabetes mellitus = 7 %,
		RR = 1.4 (1.0–2.0)			Insulin dependent diabetes mellitus = 7 %
	Insulin-dependent diabetes mellitus	RR = 1.4 (0.9–2.2)			
**Hageman et al. (2014)** [43]	CCI and age	OR = 1.1, *P* < 0.01, R^2^ = 0.03	Not investigated		2 % readmitted without surgical adverse event
					4 % readmitted with surgical adverse event
**Ling et al. (2013)** [19]	Age 60–70	Referent	Not investigated		9 %
	Age 70–80	OR = 1.60 (0.31–8.22)			
	Age 80–90	OR = 3.91 (0.83–18.4)			
	Age > 90	**OR = 7.21 (1.28–40.65)**			
	Female	Referent			
	Male	OR = 0.75 (0.27–2.10)			
	Intertrochanteric	OR = 0.84 (0.36–1.95)			
	Comorbidity = 0	Referent			
	Comorbidity > 1	OR = 0.73 (0.26–2.04)			
	Comorbidity > 2	OR = 0.48 (0.10–2.26)			
	Comorbidity > 3	OR = 1.53 (0.45–5.19)			
	Renal failure	OR = 2.49 (0.50–12.4)			
	Serum albumin	OR = 2.09 (0.69–6.36)			
	Serum iPTH	OR = 1.01 (0.42–2.47)			
	Vitamin D deficiency	OR = 1.00 (0.43–2.33)			
	Euthyroid	Referent			
	Overt hypothyroidism	OR = 1.75 (0.35–8.89)			
	Thyroid dysfunction	OR = 1.19 (0.47–3.03)			
	Subclinical hypothyroidism	OR = 0.44 (0.05–3.54)			
**Radcliff (2008)** [51]	White race	OR = 1.32	Plate/screw (CPT 27244)	OR = 1.26	7 %
	Age 65–74	Referent	Open reduction (CPT 27236)	OR = 1.13	
	Age 75–84	OR = 1.17	Hemiarthroplasty (CPT 27125)	OR = 1.30	
	Age ≥ 85	OR = 0.95	Percutaneous fixation (CPT 27235)	OR = 1.05	
	Currently smoking	OR = 0.94	Intramedullary implant (CPT 27245)	OR = 0.92	
	Alcohol use (>2 drinks/day)	OR = 1.29	General anesthesia	OR = 0.97	
	Partial independence	OR = 1.04	Blood transfusion (1 U)	OR = 1.01	
	Total independence	OR = 0.70	Surgery 4 days after admission	OR = 0.70	
	Impaired sensorium	**OR = 1.67**	Weekend surgery	OR = 1.15	
	Renal insufficiency	**OR = 1.46**	Wound not “clean”	OR = 1.44	
	Steroid use	OR = 1.10	Emergency admission	OR = 0.74	
	Disseminated cancer	OR = 0.87	ASA class 3	OR = 1.38	
	Congestive heart failure	OR = 1.28	ASA class 4 or 5	OR = 1.60	
	Dementia	OR = 0.75			
	Diabetes	OR = 1.09			
	Hemiplegia	OR = 1.02			
	Severe chronic obstructive pulmonary disease	OR = 1.24			
	Recent weight loss	OR = 0.99			
	Hyponatremia	OR = 1.73			
**Toy et al. (2014)** [41]	History of pulmonary disease	OR = 2.0	Inpatient status before procedure	OR = 1.9	10.8 %
**Tsai (2013)** [47]	Hospitalization for all reasons	OR = 0.74 (0.59–0.93)	Not investigated		Hospitalization for all reasons: 14.73 %
	Fracture related diagnoses	OR = 0.69 (0.45–1.05)			Fracture-related diagnoses: 3.73 %
	Musculoskeletal disorders	OR = 0.60 (0.37–0.98)			Musculoskeletal disorders: 2.36 %
	Hospitalization for other diagnoses	OR = 0.83 (0.62–1.12)			Hospitalization for other diagnoses: 9.23 %
***Readmission to hospital within 30 days of admission to nursing home***					
**Kiel et al. (1994)** [45]	Age 74–85	OR = 0.58 (0.40–0.83)	Not investigated		12.4 %
	Age > 85	OR = 0.55 (0.38–0.80)			
	Secondary neurological diagnoses	OR = 0.75 (0.56–1.00)			
	Living with someone	**OR = 1.44 (1.12–1.87)**			
	Any dependency in ADLs	**OR = 1.45 (1.08–1.93)**			
	Ability to walk	**OR = 1.54 (1.16–2.05)**			
**Readmission >30 days**					
***Readmission to hospital within 60 days from discharge***					
**Halm et al. (2003)** [27]	Active clinical issue in the 24 h before discharge	OR = 1.6 (1.0–2.6)	Not investigated		18.8 %
	New impairment in the 24 h before discharge	**OR = 1.7 (1.2–2.3)**			
**Halm et al. (2003)** [33]	Transfusion when Hb < 10.0 g/dL	OR = 0.52 (0.28–0.97)			16.9 %
**Halm et al. (2004)** [34]	Hb on admission	OR = 0.69 (0.49–0.95)	Not investigated		16.9 %
	Hb lowest preoperative	OR = 0.65 (0.48–0.89)			
	Hb lowest postoperative	OR = 0.78 (0.64–0.95)			
***Readmission within 80 and 180 days***					
**Ottenbacher et al. (2003)** [46]	Age	Beta = 0.943, SEM = 0.374, LR = 3.51	Not investigated		16.7 %
	Ethnicity × gender	Beta = 0.012, SEM = 0.005, LR = 2.54			
	FIM rating	Beta = −0.825, SEM = 0.293, LR = 4.86			
**Readmission within 90 days from discharge**					
**Vochteloo et al. (2011)** [22]	Age	OR = 0.97 (0.94-0.99)	ASA	OR = 1.43 (0.99–2.09)	Anemic group 12.9 %, Non-anemic group 9.0 %
	Anemia	**RR = 1.24 (1.04–1.49)**	General anesthesia	OR = 0.35 (0.13–0.99)	
**Readmission 90 days from surgery**					
**Zhang et al. (2014)** [52]	Male	HR = 0.77 (0.72–0.83)	Hemiarthroplasty	HR = 0.77 (0.71–0.83)	90 day readmission rate = 14 %
	African-American race	**HR = 1.22 (1.02–1.46)**	RTSA	HR = 0.82 (0.67–0.99)	15 % for open reduction-internal fixation and RTSA
	Medical comorbidities (per diagnosis)	**HR = 1.20 (1.18–1.22)**			13 % for hemiarthroplasty
	Insurance with Medicaid	**HR = 1.27 (1.08–1.49)**			
	Private insurance	HR = 0.82 (0.74–0.91)			
	Discharge status – home with services	**HR = 1.19 (1.07–1.32)**			
	Transfer to facility nursing or rehab	HR = 1.99 (0.82–2.18)			
**Gregersen et al. (2011)** [42]	Postop Hb levels ≤ 6 mmol/L	**OR = 3.24 (1.15–9.14)**	Intervention care	OR = 0.47 (0.23–0.94)	14 % intervention care
	Age	**OR = 2.98 (1.08–8.21)**			26 % standard care
**Readmission within 180 days from surgery**					
**Tsai (2013)** [47]	Hospitalization for all reasons	OR = 0.93 (0.78–1.38)	Not investigated		Hospitalization for all reasons: 38.31 %
	Fracture related diagnoses	OR = 0.90 (0.67–1.21)			Fracture related diagnoses: 9.14 %
	Musculoskeletal disorders	OR = 1.03 (0.77–1.38)			Musculoskeletal disorders: 9.43 %
	Hospitalization for other diagnoses	OR = 0.93 (0.77–1.13)			Hospitalization for other diagnoses: 26.72 %
**Readmissions within 6 months from discharge**					
**Härstedt et al. (2015)** [35]	Hypertension	**OR = 2.0 (1.2–1.9)**	Not investigated		32 %
	Atrial Fibrillation	OR = 0.80 (0.40–1.61)			73 % were admitted once only
	Myocardial infarction	OR = 0.70 (0.30–1.64)			
	Angina pectoris	OR = 0.49 (0.19–1.26)			
	Heart failure	OR = 0.69 (0.29–1.61)			
	Pacemaker	**OR = 6.64 (1.68–26.33)**			
	Valvular heart disease	OR = 0.87 (0.17–4.60)			
	Syncope	OR = 0.99 (0.36–2.71)			
	Stroke	OR = 0.66 (0.31–1.40)			
	Pulmonary embolism/deep vein thrombosis	OR = 2.72 (0.80–9.24)			
	Peripheral vascular disease	OR = 1.01 (0.33–3.08)			
	Parkinson’s disease	OR = 1.32 (0.32–5.70)			
	Epilepsy	OR = 0.26 (0.03–2.15)			
	Cognitive disorder (dementia)	OR = 1.68 (0.94–3.01)			
	Depression	OR = 1.54 (0.63–3.78)			
	Diabetes mellitus	OR = 0.64 (0.29–1.42)			
	Thyroid disease	OR = 1.47 (0.70–3.12)			
	Respiratory disease (COPD)	OR = 0.98 (0.42–2.26)			
	Malignancy	OR = 1.16 (0.57–2.37)			
	Autoimmune disorders	OR = 2.30 (0.87–6.10)			
	Prostate tumor (men)	OR = 4.99 (0.92–27.18)			
	Previous fracture	OR = 1.70 (0.86–3.36)			
	Osteoporosis	OR = 0.30 (0.07–1.40)			
	Diseases in the kidney and urinary tract				
		OR = 1.72 (0.57–5.16)			
	Anemia	OR = 1.19 (0.43–3.32)			
	ASA classification per one grade	OR = 1.67 (0.99–2.80)			
**Riggs et al. (2010)** [39]	Discharge to rehabilitation	Standard coeff −0.095 (−0.102 to −0.11)	LOS 75th quartile ≥ 9 days)	Standard coefficient 0.151 (0.044–0.141)	8.3 %
			Any days in Intensive Care Unit	Standard coefficient 0.168 (0.097–0.271)	
**Readmission after 12 months from discharge**					
**Bischoff- Ferrari (2010)** [20]			2000- vs. 800-IU/d dosage of cholecalciferol	Relative rate different, −39 % (−62 % to −1 %)	70 % had 1 readmission, 22 % had 2 readmissions and 7 % had 3 readmissions
			Efficacy analysis: 2000 IU/d dose	Relative rate different, −55 % (−79 % to −2 %)	
**Giusti et al. (2008)** [32]	Age 76–85	OR = 0.77 (0.29–2.01)	Not investigated		30.1 %
	Age > 85	OR = 0.46 (0.16–1.29)			
	CIRS-SI 1.5–1.9	**OR = 5.95 (1.66–21.3)**			
	CIRS-SI > 1.9	**OR = 7.05 (1.68–29.7)**			
	2 month ADL Katz Index 0–2	**OR = 3.02 (1.09–8.32)**			
**Heyes et al. (2015)** [29]	Female	OR = 1.34 (0.65–2.76)	Time to surgery 36 h to 6 days	OR = 1.62 (0.156–2.44)	44 %
	Cephalomedullary nail	OR = 1.51 (0.40–1.08)	>6 days	OR = 1.29 (0.198–3.02)	
	Hip hemiarthroplasty/THR	OR = 3.10 (0.19–1.80)	Inpatient stay > 7 days	**OR = 3.13 (0.12 –0.62)**	
	Moderate alcoholic	OR = 1.36 (0.31–1.73)	Inpatient stay of 7–14 days	**OR = 7.04 (0.05 –0.34)**	
	Alcoholic	OR = 1.52 (0.26–1.66)	Inpatient stay of 14–21 days	**OR = 2.90 (0.18 –0.64)**	
	Affected side-right	OR = 1.10 (0.57–1.45)	Inpatient stay of 21–28 days	OR = 1.83 (0.25–0.16)	
	Ex-smoker	OR = 1.14 (0.64–2.00)	Inpatient stay of 28–35 days	OR = 2.11 (0.19–1.17)	
	Smoker	OR = 1.24 (0.56–2.72)	ASA score > 2	OR = 3.68 (0.06–1.15)	
	Residential care/nursing home residence	**OR = 1.71 (1.34–1.98)**	ASA score > 3	OR = 1.95 (0.17–1.48)	
			ASA score > 4	OR = 2.14 (0.16–1.33)	
	Hb > 2 g/dL drop	OR = 1.29 (0.48–1.24)	Transfusion status < 2 units	OR = 1.12 (0.31–4.00)	
	Admission glucose > 7.8 mmol/L	OR = 1.18 (0.66–2.09)	Transfusion status > 2 units	OR = 1.85 (0.48–7.04)	
	Discharge glucose > 7.8 mmol/L	OR = 1.05 (0.53–1.70)			
	Total protein	OR = 1.13 (0.53–1.46)			
	Admission eGFR < 45	OR = 1.04 (0.50–1.83)			
	Discharge eGFR < 45	OR = 1.04 (0.47–1.96)			
**Hsaio et al. (2011)** [23]	Long-term use of alendronate reduces risk	HR = 0.27 (0.15–0.78)	Not investigated		8.6 % cases untreated cohort; 6.3 % alendronate users; 7.6 % other anti organophosphorous drug users
**Intrator et al. (1998)** [44]	Home healthcare usage	OR = 0.77 (0.52–1.15)	Not investigated		Rehab only group 34.1 % Rehab and home health group 27.2 %
**Jou et al. (2014)** [24]	1 year:		1 year: District hospital	**HR = 2.24 (1.82–2.75)**	50–74 y (6.02 %)
	Age < 75 Age ≥ 75	Referent	LOS		≥75 y (8.38 %)
		**HR = 1.46 (1.24–1.73)**	≤10 days ≥11 days	Referent	
	1 year: CCI = 0 CCI score ≥ 2	Referent		HR = 0.51 (0.43–0.60)	
		**HR = 1.28 (1.09–1.51)**	1 year: Geographic regions		
			Northern Central Southern Eastern	Referent	
				HR = 1.12 (0.90–1.39)	
				HR = 1.07 (0.88–1.29)	
				HR = 0.89 (0.54–1.46)	
**Merchant (2005)** [38]	Post-operative complications	After adjustment for potential covariates the presence of postoperative complications was not significant (*P* > 0.05, coefficients not presented)	Not investigated		31.7 %
**Teixeira et al. (2009)** [40]	Male (predicts related first readmission)	**HR = 1.25 (1.08–1.46)**	Teaching hospital vs. public hospital (predicts related first readmission)		32 %
	Male	**HR = 1.36 (1.16–1.59)**		HR = 0.86 (0.79–0.95)	
	Increasing age (predicts unrelated first readmission)	HR = 0.94 (0.89–0.99)	Index stay in a private hospital	HR = 0.78 (0.67–0.9)	
	Cancer	**HR = 1.41 (1.03–1.94)**	Teaching hospital (predicts unrelated first readmission)	HR = 0.87 (0.79–0.95)	
	Kidney disease	HR = 1.38 (1.00–1.90)			
	Dementia (predicts related first readmission)	**HR = 1.21 (1.01–1.46)**			
	Dementia (predicts unrelated first readmission)	HR = 0.68 (0.53–0.87)			
**Readmission within 18 months from discharge**					
**Fox et al. (1998)** [31]	Performance on balance tests at 2 months post fracture	**Beta = −0.155,** ***P*** **= 0.01**			
	Gait score	Beta = −0.013, *P* = 0.83			
	Mobility score	Beta = −0.098, *P* = 0.11			

CCI, Charlson comorbidity index; CIRS, Cumulative illness rating scale – severity index; ASA, American Society of Anesthesiologists score; LOS, length of stay; ADL, activities of daily living; FIM, functional independence measure; CM, conservative treatment; RTSA, reverse total shoulder arthroplasty; Hb, hemoglobin; HR, hazards ratio; OR, odds ratio; LR, likelihood ratio; RR, relative risk

^a^The term readmission is being used as the studies have reported on hospital readmissions rather than hospital re-presentations

Bold text indicates a significant association (*p* <0.05)

#### Patient characteristics

Patient characteristics that were investigated as possible risk factors were age, gender, physical function, and level of independence with daily living. Seven of the 14 studies that investigated age reported a significant positive association [19, 21, 24, 28, 30, 36, 49]. Six studies examined the effect of male sex on subsequent hospital readmission, and three found male sex to be a risk factor of readmission [25, 30, 40]. Two studies reported being aged > 75 years and receiving treatment from a regional hospital for the index hospital event as predictors of hospital readmissions at 14 days, 30 days, and 1 year after the index event [24, 47]. A study that examined predictors of hospital readmissions within 1 year of discharge identified male gender and increasing age as risk factors of hospital readmissions [40]. Four out of five studies that examined the Cumulative Illness Rating Score (CIRS) identified that a CIRS score > 2 was predictive of hospital readmission [21, 24, 28, 32]. Five studies that investigated residential status of patients after the index hospital event found a positive correlation between discharge to a nursing home and 30-day risk of hospital readmission [29, 30, 36, 45, 50].

Physical and mental health comorbidities were also examined as potential risk factors for readmissions; there was, however, a considerable variation in the comorbidities investigated. Eight studies examined the association between cardiovascular disease and hospital readmission: five of the studies found a positive association [25, 28, 30, 49, 50]. Eight studies examined the association between diabetes and readmission. Three of these studies reported a significant positive association [25, 36, 49], but two that only included surgical cases did not find an association. Two of the five studies that investigated renal insufficiencies and kidney diseases as predictors of readmission reported significant positive associations [25, 51]. One of the three studies that examined post-surgical anemia and one of the four studies that specifically examined hemoglobin (Hb) reported a significant positive association (Hb < 6 mmol/L) with hospital readmission within 90 days [42]. One study identified cancer and dementia as comorbidities at the index event to be predictive of hospital readmission within a year [40]. One study examined body mass index (BMI) and reported that patients with a BMI > 35 were at an elevated risk of being readmitted to hospital after discharge [30]. Among the cognitive disorders, dementia was the most common comorbidity examined and was positively associated with readmissions in three of the six studies in which it was investigated [28, 40, 49].

In total, comorbidities were significant risk factors and reasons for hospital readmission in 20 studies. The most common comorbidities identified were myocardial infarction (*n* = 9) [25, 28, 35, 36, 40, 41, 48, 51, 53], pulmonary embolism (*n* = 7) [25, 28, 39–41, 51, 53], urinary tract infection (*n* = 6) [36, 38, 41, 48, 50, 51], pneumonia (*n* = 9) [20, 29, 36, 38, 41, 42, 48, 50, 51], sepsis (*n* = 5) [20, 36, 41, 48, 51], and renal failure (*n* = 4) [36, 41, 48, 53]. Other frequent reasons for readmission included surgical complications (*n* = 6) [28, 40, 41, 43, 50, 52], re-fractures (*n* = 5) [24, 28, 42, 50], and falls (*n* = 3) [35, 36, 38].

#### Other clinical and hospital indicators

A range of other clinical and hospital factors were examined. Length of stay in hospital served as a predictor of re-presentation in six studies; of these, five studies reported that a longer length of stay increased the risk of subsequent hospital readmissions [24, 25, 29, 50]. An American Society of Anesthesiologists (ASA) score > 3 was positively associated with risk of hospital readmission [30] in one of the four studies in which it was investigated. In another study, surgical delay of 24 hours or more was associated with readmission [28]. One study observed that older adults admitted into a geriatric unit managed by a multidisciplinary team had lower risk of hospital readmission and improved walking ability [21].

#### Quality assessment

Findings from the quality assessment of the studies are presented in Table 4. The global rating score for most studies (*n* = 17; 48 %) was in the ‘moderate’ category. However, the quality of 11 of the 35 studies (31 %) was classified as ‘strong’. All 11 strong studies examined patients with hip fractures. Another seven studies (7 %), which examined older adults with hip fractures, received a score of ‘weak’. The weaknesses most frequently identified were a failure to report drop outs or withdrawals, a lack of clear explanation about data collection processes, and inadequate descriptions of how potential confounders were controlled for.

**Table 4:** Quality assessment classifications from the Effective Public Health Practice Project Quality Assessment Tool

Lead author	Year	Selection bias	Study design	Confounder	Blinding	Data collection	Dropouts & withdrawals	Global rating
Basques	2015 [30]	Moderate	Moderate	Moderate	Moderate	Strong	Weak	Moderate
Bischoff-Ferrari	2010 [20]	Weak	Strong	Strong	Strong	Moderate	Strong	Strong
Boddaert	2014 [21]	Moderate	Moderate	Strong	Moderate	Strong	Strong	Strong
Fox	1998 [31]	Weak	Moderate	Weak	Moderate	Strong	Weak	Weak
French	2008 [25]	Moderate	Moderate	Weak	Moderate	Strong	Strong	Moderate
Giusti	2008 [32]	Strong	Moderate	Weak	Moderate	Strong	Strong	Moderate
Golinvaux	2014 [48]	Moderate	Moderate	Strong	Moderate	Strong	Strong	Strong
Gregersen	2011 [42]	Moderate	Moderate	Strong	Moderate	Strong	Strong	Strong
Hageman	2014 [43]	Moderate	Moderate	Weak	Moderate	Strong	Weak	Weak
Halm	2003 [27]	Strong	Moderate	Strong	Moderate	Strong	Strong	Strong
Halm	2003 [33]	Strong	Moderate	Strong	Moderate	Strong	Strong	Strong
Halm	2004 [34]	Strong	Moderate	Strong	Moderate	Strong	Strong	Strong
Härstedt	2015 [35]	Strong	Moderate	Weak	Moderate	Strong	Strong	Moderate
Heyes	2015 [29]	Moderate	Moderate	Weak	Moderate	Strong	Moderate	Moderate
Heidari	2012 [26]	Moderate	Moderate	Strong	Moderate	Strong	Weak	Moderate
Hsaio	2011 [23]	Moderate	Moderate	Strong	Moderate	Weak	Weak	Weak
Intrator	1998 [44]	Weak	Moderate	Strong	Moderate	Strong	Strong	Weak
Jou	2014 [24]	Moderate	Moderate	Strong	Moderate	Weak	Weak	Weak
Kates	2014 [28]	Moderate	Moderate	Weak	Moderate	Strong	Strong	Moderate
Kates	2015 [49]	Moderate	Moderate	Weak	Moderate	Strong	Strong	Moderate
Khan	2012 [36]	Moderate	Moderate	Weak	Moderate	Strong	Moderate	Moderate
Kiel	1994 [45]	Moderate	Moderate	Weak	Moderate	Strong	Strong	Moderate
Le-Wendling	2012 [37]	Moderate	Moderate	Strong	Moderate	Weak	Strong	Moderate
Ling	2013 [19]	Moderate	Moderate	Moderate	Moderate	Strong	Weak	Moderate
Merchant	2005 [38]	Moderate	Moderate	Moderate	Moderate	Strong	Strong	Strong
Ottenbacher	2003 [46]	Moderate	Moderate	Weak	Moderate	Strong	Moderate	Moderate
Pollock	2015 [50]	Moderate	Moderate	Weak	Moderate	Strong	Weak	Weak
Radcliff	2008 [51]	Moderate	Moderate	Strong	Moderate	Strong	Strong	Strong
Riggs	2010 [39]	Moderate	Moderate	Moderate	Moderate	Strong	Weak	Moderate
Teixeira	2009 [40]	Moderate	Moderate	Moderate	Moderate	Strong	Strong	Strong
Toson	2015 [53]	Moderate	Moderate	Moderate	Moderate	Strong	Moderate	Strong
Toy	2014 [41]	Moderate	Moderate	Weak	Moderate	Strong	Strong	Moderate
Tsai	2013 [47]	Moderate	Moderate	Strong	Moderate	Weak	Weak	Moderate
Vochteloo	2011 [22]	Moderate	Moderate	Strong	Moderate	Strong	Weak	Moderate
Zhang	2014 [52]	Moderate	Moderate	Weak	Moderate	Moderate	Weak	Weak

#### Best-evidence synthesis

Eleven studies met the inclusion criteria for high quality studies. In accordance with the global rating scale, these studies had no ‘weak’ ratings in any sub-domain (Table 4). Five of these studies (45 % of high quality studies) reported at least one statistically significant risk factor of hospital readmission that was identifiable at the index admission [21, 27, 40, 42, 53]. Among the patient factors associated with readmission in these five studies, age was positively associated with hospital readmission in one study [21]. One study each out of the 11 high quality studies identified male sex, lower post-operative Hb level, and higher CIRS score at index admission to have positive associations with hospital re-presentations [21, 40, 42]. Comorbidities that were significantly associated with hospital re-presentations in these studies included impaired sensorium, renal insufficiencies, asthma, chronic liver disease, dementia, cancer, ‘new impairments’ on discharge, adverse effects of glucocorticoids, and androgen therapy [21, 27, 40, 42, 51]. In summary, of the 11 high quality studies (31 % of all included studies), five provided evidence of statistically significant findings, and the correlates that were significant varied among studies.

#### Meta-analysis

**Fig. 2:**
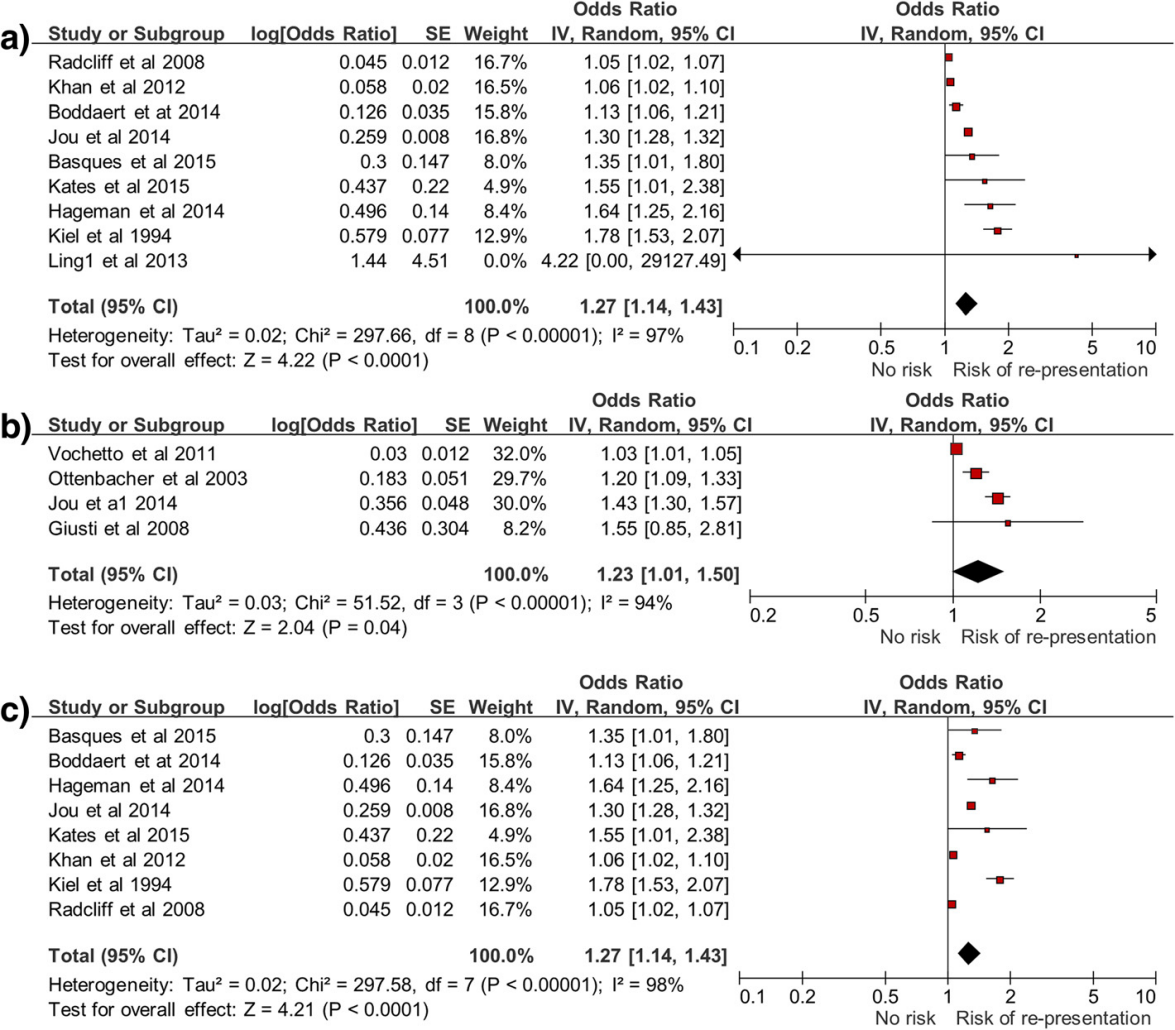
Forest plot of age as a predictor of hospital re-presentation within 30 days (**a**), after 30 days (**b**), and sensitivity analysis (**c**) (within 30 days)

The meta-analysis indicated age was associated with increased risk of hospital readmission both within a 30-day time-frame and beyond a 30-day time-frame (Fig. 2), with the 95 % CIs of the pooled effect estimate not inclusive of 1.00. The random-effects pooled OR was 1.27 (95 % CI, 1.14–1.43) for the effect of age on the risk of hospital readmission within 30 days (Fig. 2a). However, a large amount of heterogeneity (I^2^ = 98 %) in study effect size estimates was observed. The random-effects pooled OR was 1.23 (95 % CI, 1.01–1.50) for the effect of age on the risk of hospital readmission > 30 days (Fig. 2b). The heterogeneity was also large (I^2^ = 94 %) among studies reporting hospital readmission > 30 days. The sensitivity analysis indicated that the removal of the small study [19] with an outlying effect estimate had no difference on the pooled effect estimate (Fig. 2c) and had a negligible effect on overall heterogeneity (I^2^ = 97 %). It is noteworthy that the calculations that were required to determine pooled effect estimates from studies with disparate analysis and reporting approaches resulted in pooled ORs that cannot be interpreted as simple effects per increasing year of age. However, the findings of an increasing risk with age, the demonstrated significance at a 95 % CI, and the substantial variation in reported effect among studies were noteworthy findings from the meta-analysis.

## Discussion

There are a number of useful inferences and research priorities that can be drawn from the findings reported in this review. A key finding was that age was the most frequently investigated risk factor for hospital readmission. The meta-analysis confirmed age as a predictor of hospital re-presentations both within 30 days and for re-presentations occurring after 30 days. Although age is not modifiable, interventions that target high-risk older adults before they leave hospital have been cost-effective in reducing undesirable outcomes, and it has been suggested that there may be some utility for these interventions to be offered to older people recovering from fragility fractures [54, 55]. An important consideration for future research investigating age as a predictor of hospital re-presentations may be to consider the linearity of the effect of age on risk of re-presentation to hospital. The risk of readmission may not increase uniformly with increasing age in years, but rather, there may be an accelerating increase in risk of readmission with advancing age among people recovering from fragility fractures. However, further research is required to confirm or refute this hypothesis in the context of older adults recovering from fragility fractures.

There was a high degree of variation (methodologies, reporting quality, and results) across studies reporting other potential risk factors. A salient finding from this review was that studies with a high quality rating reported the following factors, which were identified at the index admission, to be significant predictors of re-presentation to hospital: higher CIRS, ASA > 3, cardiovascular diseases, low post-operative Hb, kidney diseases, dementia, and cancer [21, 27, 40, 42, 51]. Other potential predictors identified from studies with a moderate quality rating included anemia, neurological disorders, delirium, renal failure, diabetes, longer length of stay, and being discharged to a residential nursing care facility [22, 25, 28, 36]. Like age, many of these risk factors are likely to be difficult to modify in the context of clinical care during a hospitalization. However, they may prove useful for guiding the delivery of appropriate (and potentially targeted) care models to offset this risk. Co-morbidities and length of stay, which were reported as potential risk indicators in the present review, are generally consistent with research among other clinical populations [13, 56, 57]. This is a useful finding, so far as it implies that interventions to reduce re-presentations that have been successful among other clinical populations are worthy of consideration for adaptation and evaluation, specifically among patients with fragility fractures.

It was interesting to note that no factor that was investigated in multiple studies was consistently associated with readmission in all studies in which it was investigated. This observation of inconsistency among studies for the same risk factor may seem innocuous, but in actuality highlights one of the key challenges in the field. The inconsistency may be attributable to genuine variation in risk factors between populations and dissimilar health services; however, it may be attributable to methodological and reporting inconsistencies among studies that may have contributed to seemingly incongruent findings. This review has highlighted the extent of these inconsistencies among studies in a systematic way for the first time and should act as a call to reduce unnecessary variation between health services and research methodologies in this field.

Perhaps of even greater importance than potential inconsistencies in findings was the gap in the literature revealed in this systematic review. Specifically, a novel finding was that no study was identified that had examined risk factors for re-presentation to ED without hospital admission. Older adults disproportionately consume ED resources and have been reported to account for 20 % of presentations to EDs [58, 59]. This absence of studies examining re-presentations to EDs without admission to hospital by patients recovering from fragility fractures represents an important gap in the literature worthy of further research to advance the field.

It was also notable that most of the 35 studies focused on people treated for a hip fracture, including the eleven studies with highest quality ratings [20, 21, 27, 33, 34, 38, 40, 42, 48, 51, 53]. Identifying the paucity of high quality studies that have examined risk factors for re-presentation to hospital following fragility fractures that affect other important body regions (e.g., spine, shoulder, pelvis (non-hip), ankle, wrist, and forearm) is another important finding from this review. Nonetheless, this review has provided a consolidated synthesis of risk factors for hospital re-presentations taking into account study quality and consistency (and inconsistencies) among studies.

### Strengths and limitations

A major strength of this review was that it used broad search terms and multiple databases. A rigorous screening process was implemented, including two researchers to independently conduct each stage of screening, data extraction, and quality appraisal. The investigators also considered it beneficial to have used the same quality measurement tool that could be applied across a range of study designs. This reduced the potential for quality rating bias attributable to use of differing quality rating instruments for different study designs. Along with the aforementioned strengths were some notable limitations of this review. First, the review was restricted to peer-reviewed journal articles published in the English language. Second, the inclusion of a range of study designs, sample characteristics, and lengths of study enrolment periods contributed to heterogeneity that prohibited the valid pooling of data for meta-analyses for most potential predictors. This was compounded by other methodological and reporting differences across studies.

## Conclusions

There are several important recommendations for future research following this investigation. First, further robust examinations of risk factors for re-presentation to hospital among patients who have sustained fragility fractures beyond those affected by hip fractures are warranted. Second, investigation of risk factors for ED re-presentation without admission are also worthy of investigation. Understanding risk factors for these re-presentations may inform service enhancement to reduce the need for these patients to present to a hospital ED. Third, investigations into how specific elements of geriatric clinical care models potentially related to risk of re-presentation can be optimized to reduce risk would be beneficial. While some differences in findings among studies may be attributable to study methodology, it is likely that other discrepancies were due to local clinical, patient, or environmental factors. A greater understanding of the reasons for variations in risk factors across geographical locations, services, and patient samples may inform the development of interventions or alternative models of care for improving patient care and reducing risk.

A further pragmatic consideration is that the use of emergency services and readmissions to hospitals other than where the primary admission took place ought to be considered wherever possible. Moreover, consistency in the categorization of variables (e.g., age), definition of the index event (e.g., date of discharge), and follow-up periods (e.g. 30, 60, and 90 days) would be beneficial for comparability across studies.

## Abbreviations

ASA: American Society of Anesthesiologist
CCI: Charlson comorbidity index
CIRS: cumulative illness rating score
ED: emergency department
HR: hazard ratio
LOS: length of stay
OR: odds ratio
